# The impact of a brief psychoeducational intervention on hospital security personnel’s attitudes towards aggression and violence management at Cecilia Makiwane Hospital

**DOI:** 10.4102/sajpsychiatry.v32i0.2648

**Published:** 2026-08-21

**Authors:** Inathi Mncinci, Yanga Thungana

**Affiliations:** 1 Department of Psychiatry, Faculty of Health Sciences, Walter Sisulu University, Mthatha, South Africa; 2 Department of Psychiatry, Faculty of Health Sciences, Nelson Mandela University, Gqeberha, South Africa

**Keywords:** aggression management, hospital security personnel, de-escalation training, biopsychosocial model, South Africa

## Abstract

**Background:**

Aggression and violence remain persistent challenges in healthcare settings, particularly in psychiatric and emergency units. Security personnel serve as frontline responders but frequently lack structured training in de-escalation techniques, increasing the risk of harm.

**Aim:**

This study aimed to assess and improve security personnel’s attitudes towards the causes and management of aggression through a structured de-escalation training programme.

**Setting:**

The study was conducted at Cecilia Makiwane Hospital, a regional academic facility in the Eastern Cape province of South Africa, housing the only public-sector mental health unit in Buffalo City Municipality.

**Methods:**

A quasi-experimental single-group pre-test–post-test design was used. Security personnel completed an interactive educational programme incorporating discussions, role-play, and scenario-based exercises. The Management of Aggression and Violence Attitude Scale (MAVAS) was administered before and after the intervention. Data were analysed using paired-samples *t*-tests and multiple regression at a 5% significance level.

**Results:**

Baseline MAVAS domain scores indicated moderate agreement with all four domains. Following the intervention, significant improvements were observed across all MAVAS domains (*p* ≤ 0.01), reflecting more positive attitudes towards aggression management. Female participants showed greater changes in environmental and interpersonal causes of aggression (*B* = −0.259, *p* = 0.003), while longer work experience predicted increased recognition of patient-related causes. Job rank and age were not significant predictors.

**Conclusion:**

Structured de-escalation training effectively improved security personnel’s attitudes towards managing aggression and violence in a hospital setting.

**Contribution:**

This study provides empirical evidence that context-specific de-escalation training for security personnel may support workplace safety and strengthen multidisciplinary collaboration in the South African setting.

## Introduction

Aggression and violence in healthcare settings are widely recognised as significant global occupational and public health concerns, posing risks to the safety and wellbeing of both healthcare workers and patients.^1,2,3,4,5^ Such incidents are particularly prevalent in psychiatric and emergency care environments, where patients may present with acute agitation, intoxication, delirium, or severe psychological distress.^6,7^

In South Africa, early studies conducted in the late 1990s and early 2000s documented high levels of aggression and violence directed towards healthcare workers, especially within psychiatric and emergency settings.^8,9^ Although these studies provide important historical context, more recent national and provincial data indicate that workplace violence remains a persistent challenge in South African public healthcare facilities, particularly in under-resourced settings.^10,11^ These ongoing challenges reflect broader societal patterns of violence and contribute to staff injury, emotional distress, burnout, and disruption of care delivery.^11,12^

Hospital security personnel play a critical role in maintaining safety within healthcare environments and are often among the first responders to aggressive or violent incidents. Despite this responsibility, security staff frequently receive little or no formal training in aggression management or de-escalation techniques.^13,14,15^ Inadequate preparation may result in responses that unintentionally escalate situations, increase reliance on coercive measures, and compromise therapeutic engagement.^14,16,17,18^ In addition, poorly managed incidents may perpetuate stigma, undermine patient dignity, and conflict with human rights–based approaches to mental healthcare.^13,19,20,21^

International literature consistently demonstrates that structured de-escalation training can improve staff attitudes towards aggression, enhance confidence, reduce reliance on physical restraint, and promote safer clinical environments.^22,23,24,25,26,27,28^ However, the majority of these studies have focused on clinical staff in high-income countries. Evidence addressing the preparedness, attitudes, or training needs of hospital security personnel – particularly within low- and middle-income settings – remains limited.^19,20,21,29,30^

Within the South African context, there is a notable lack of empirical research examining hospital security personnel’s attitudes towards aggression or their exposure to structured de-escalation training. Consequently, many security officers operate without standardised guidance or evidence-informed frameworks, leading to inconsistent and reactive approaches to managing aggression.^13,14,15,16,17,18,31^

This study is informed by the biopsychosocial model of aggression, which conceptualises aggressive behaviour as arising from the interaction of biological, psychological, and social factors.^19,32,33,34^ Within this framework, aggression is viewed as potentially preventable through early recognition of triggers and the application of non-coercive, therapeutic interventions. De-escalation theory complements this model by emphasising calm communication, empathy, respect, and situational awareness as central strategies for preventing escalation and maintaining safety.^22,23,24,27,35^

Cecilia Makiwane Hospital (CMH), a large regional facility in the Eastern Cape province, reflects many of the systemic challenges faced by public hospitals in South Africa, including frequent incidents of patient aggression and limited access to structured training for security personnel.^10,13,16^ Security officers at this site routinely intervene in high-risk situations despite limited formal preparation, underscoring the need for targeted educational interventions.

Therefore, this study aimed to assess and improve hospital security personnel’s attitudes towards the causes and management of aggression and violence through a structured de-escalation training programme. Using the Management of Aggression and Violence Attitude Scale (MAVAS), the study evaluated baseline attitudes, implemented an evidence-informed educational intervention, and examined changes in attitudes following training. By focusing on an underexplored but critical group within the healthcare workforce, this study seeks to contribute evidence to inform future training initiatives, policy development, and violence prevention strategies in South African healthcare settings.

## Research methods and design

### Study design

This study employed a quasi-experimental pre-test–post-test design conducted among security personnel at CMH. The design involved a single group of participants who were assessed before and after a structured educational intervention, without a control group. This approach was suitable for evaluating the impact of training in a real-world hospital setting, where randomisation was not feasible.

### Setting

The study was conducted at CMH, located in Mdantsane, Buffalo City Metropolitan Municipality, Eastern Cape province, South Africa. Cecilia Makiwane Hospital is a regional academic hospital affiliated with Walter Sisulu University and Lilitha College of Nursing, providing a wide range of clinical and allied health services, including psychiatry, emergency medicine, surgery, internal medicine, obstetrics, paediatrics, and trauma care.

The hospital houses the only public-sector mental health unit serving the East London region, receiving frequent referrals of aggressive and violent patients from community clinics, correctional facilities, and law enforcement agencies. While private mental health facilities exist within the broader region, this study focused exclusively on the public healthcare sector, where resource constraints and patient volumes present unique challenges for the management of aggression and violence. Security personnel at CMH are responsible for maintaining safety and preventing violence, and they play an important role in containing incidents of aggression, thereby making the hospital an appropriate site for this intervention.

### Study population and sampling strategy

The study targeted all security personnel employed at CMH (*n* = 170). A census-based convenience sampling approach was employed, whereby all eligible and available security personnel were invited to participate in the data collection. This approach was appropriate given the finite size of the workforce and the exploratory nature of the intervention.

Both newly employed and more experienced security personnel were eligible for inclusion. Years of work experience were captured as a demographic variable and categorised to assess baseline attitudes and examine whether experience influenced post-intervention changes.

A formal sample size calculation was not performed, as the intention was to recruit the entire accessible population of security personnel at the study site. The final sample size was therefore determined by staff availability, operational requirements, and willingness to participate. Security personnel at the hospital are responsible for protecting hospital property, ensuring the safety of staff and patients, and responding to incidents involving aggression or violence.

Of the total workforce, 80 security personnel did not participate: 3 were off sick during the data collection period, 12 were unavailable because of shift patterns, 5 were unavailable because of operational demands, and 60 declined to participate. Consequently, 90 participants were enrolled and completed both the pre- and post-intervention assessments.

### Intervention

The educational programme was informed by established international literature and clinical guidelines on the non-pharmacological management of aggression in healthcare settings. Core components of the intervention were grounded in the biopsychosocial model of aggression and evidence-based de-escalation frameworks, which emphasise early identification of agitation, verbal de-escalation, empathetic communication, and minimisation of coercive practices. These principles align with the National Institute for Health and Care Excellence (NICE) guideline on violence and aggression,^22^ the consensus statement on verbal de-escalation by Richmond et al.,^25^ and thematic syntheses of effective de-escalation techniques described by Price and Baker^23^ and Price et al.^27^ The overall structure and focus on non-coercive management strategies are further supported by systematic reviews evaluating de-escalation training in healthcare settings.^24,28,33^

The programme was delivered over approximately 4 h and conducted on two separate days to accommodate alternating work shifts without disrupting hospital operations. Training was facilitated by the principal investigator, a psychiatry registrar with a Diploma in Mental Health, under the supervision of a consultant psychiatrist.

The intervention comprised two structured phases, guided by a standardised training pamphlet. Phase 1 focused on understanding the nature, causes, and progression of aggressive behaviour. Participants were introduced to different forms of violence, the anger escalation cycle (incident, escalation, crisis point, and recovery), and early warning signs of aggression, including verbal threats, intimidating posture, and behavioural cues. Emphasis was placed on differentiating behaviour from the individual, recognising situational triggers, and interpreting the social and environmental context in which aggression occurs.

Phase 2 focused on the practical application of de-escalation strategies. Participants were trained in calm verbal communication, active listening, validating emotions, empathy, and respectful engagement, as well as awareness of nonverbal cues, including tone, body posture, and physical proximity. Guidance was also provided on recognising situations in which de-escalation may be unsafe or ineffective, such as severe agitation or an imminent risk of harm. The overarching objectives were to prevent escalation, stabilise potentially volatile situations, and reduce reliance on coercive or physical interventions.

Teaching methods included brief didactic presentations, facilitated group discussions, scenario-based exercises, and role-play demonstrations to promote experiential learning and the integration of skills. These methods were selected in accordance with adult learning principles and prior evaluations of de-escalation training in healthcare environments.^25,27^

### Data collection

Data were collected using the MAVAS, a validated instrument developed to assess staff attitudes towards the causes and management of aggression in healthcare settings. The MAVAS has demonstrated acceptable construct validity and internal consistency, with reported Cronbach’s alpha coefficients ranging from approximately 0.60 to 0.80 across its domains.^12^ The instrument has been widely applied in international studies involving healthcare professionals and support staff, supporting its reliability and applicability across diverse clinical settings. The MAVAS consists of 30 items grouped into four subscales: patient-related causes (8 items), environmental and interpersonal causes (7 items), ward practices and systems (8 items), and management of aggression (7 items). Each item is rated on a four-point Likert scale ranging from 1 (strongly agree) to 4 (strongly disagree).

The MAVAS was administered in both English and isiXhosa. The isiXhosa version was developed through forward translation and review by bilingual mental health professionals to ensure conceptual equivalence and clarity. Formal psychometric validation of the isiXhosa version has not yet been conducted and is acknowledged as a limitation.

Data were collected during scheduled training sessions at CMH. Eligible security personnel were approached during duty hours and provided with verbal and written information about the study. Written informed consent was obtained from all participants prior to data collection.

Baseline data were collected immediately before the educational intervention using a self-administered questionnaire. The questionnaire consisted of two sections: a demographic data form and the MAVAS. Participants completed the questionnaires individually in a designated training venue to ensure privacy and minimise distractions. The principal investigator was present to provide clarification if required, but did not influence responses.

Following completion of the baseline assessment, participants underwent the structured de-escalation training intervention. The MAVAS was re-administered to the same participants immediately after the intervention using identical procedures. Pre- and post-intervention questionnaires were linked using unique study codes to preserve anonymity while enabling paired analysis.

All completed questionnaires were reviewed for completeness at the time of collection, securely stored, and later entered into an electronic database for statistical analysis.

Prior to the main study, a brief pilot assessment was conducted among a small group of security personnel who were not included in the final sample. The pilot aimed to evaluate the clarity, feasibility, and comprehension of the questionnaire, including the isiXhosa version. No modifications were required following the pilot assessment, and pilot data were excluded from the final analysis.

Subscale scores were calculated as the mean of items within each domain, with lower scores indicating stronger agreement with statements reflecting patient-centred, contextual, and non-coercive approaches to aggression management. Changes in mean subscale scores between pre- and post-intervention assessments were used to evaluate the impact of the educational programme.

### Data analysis

Data were captured, checked for completeness, and cleaned before analysis. Statistical analysis was performed using IBM SPSS Statistics for Windows, v27.0 (IBM Corp., Armonk, New York, US). Descriptive statistics were used to summarise participant demographics and baseline characteristics.

Changes in attitudes across MAVAS domains were assessed using paired-samples *t*-tests with a two-tailed alpha of 0.05 and 95% confidence intervals (CIs). For non-normally distributed data, the Wilcoxon signed-rank test was applied as a sensitivity analysis.

Multiple linear regression analysis was conducted to identify predictors of change in MAVAS domain scores. All a priori selected demographic variables (age, sex, job rank, and years of work experience) were entered simultaneously into the regression models, regardless of their statistical significance in bivariate analyses, to control for potential confounding and ensure theoretical relevance.

### Ethical considerations

Ethical clearance to conduct this study was obtained from the Walter Sisulu University Health Research Ethics Committee (Ref. No. 029/2025). The research protocol was submitted to the National Health Research Database and received approval. Institutional permission was granted by Cecilia Makiwane Hospital management and the contracted security company. Participation was voluntary, and written informed consent was obtained before data collection. Confidentiality and anonymity were maintained using unique participant codes, and electronic data were password-protected. No financial incentives were offered, although refreshments and certificates of participation were provided.

Participation was voluntary, and written informed consent was obtained from all participants before data collection. Confidentiality and anonymity were maintained by using unique participant codes rather than personal identifiers. Data were stored securely, and all electronic files were password-protected. No financial incentives were offered, although participants received refreshments and a certificate of participation.

## Results

### Participants’ characteristics

A total of 90 participants were included in the study (Table 1). The mean age of respondents was 39.7 years (SD = 7.8). More than half were female (55.6%), and the majority were junior staff (86.7%). Most participants (54.4%) had more than 5 years of work experience. Participants were drawn from several hospital departments, including psychiatry wards, emergency (casualty) units, outpatient departments, and other high-risk areas for patient aggression.

**TABLE 1:** Participants’ characteristics (*N* = 90).

Variable	*n*	%
**Gender**		
Female	50	55.6
Male	40	44.4
**Ranking**		
Junior	78	86.7
Senior	12	13.3
**Years of working**		
1–5	41	45.6
> 5	49	54.4
**Ward**		
Casualty	6	6.7
Gate	24	26.7
OPD	4	4.4
Psychiatry male ward	3	3.3
Psychiatry female ward	10	11.1
Non-psychiatry ward	18	20.0
**Other**	25	27.8

OPD, outpatient department.

### Intervention effects on attitudes towards aggression

Baseline (pre-intervention) MAVAS domain scores indicated moderate agreement with statements relating to patient-related, environmental, and systemic contributors to aggression, as well as approaches to managing aggressive behaviour. These findings reflect participants’ initial attitudes towards aggression management prior to the educational intervention.

To determine the effect of the educational intervention, MAVAS domain scores were compared before and after training. The intervention produced statistically significant reductions across all MAVAS domains (*p* ≤ 0.01), indicating improved attitudes towards recognising and managing aggression.

The paired-samples *t*-test showed significant reductions in mean scores across all MAVAS domains after the intervention (Table 2). As MAVAS uses a four-point Likert scale (1 = strongly agree to 4 = strongly disagree), a lower post-test score indicates stronger agreement with the items in each domain. In the patient-related causes of aggression domain, the mean score decreased from 2.29 (SD = 0.44) before the intervention to 2.13 (SD = 0.38) after the intervention (*p* = 0.001), indicating increased agreement that aggression can be linked to patient-related factors. The environmental and interpersonal causes domain declined from 2.06 (SD = 0.35) to 1.89 (SD = 0.36) (*p* < 0.001), indicating increased recognition of contextual factors contributing to aggression. In the ward practices and systems domain, mean scores dropped from 2.24 (SD = 0.31) to 2.08 (SD = 0.36) (*p* < 0.001), reflecting stronger agreement that institutional and procedural influences impact aggression. Lastly, the management of aggression domain decreased from 1.89 (SD = 0.38) to 1.79 (SD = 0.32) (*p* = 0.010), suggesting more positive attitudes towards strategies for managing aggression.

**TABLE 2:** Pre- and post-intervention Management of Aggression and Violence Attitude Scale domain scores.

Domain	Pre-test mean (± SD)	Post-test mean (± SD)	Mean difference	95% CI of the difference	*p*-value
Patient-related causes of aggression	2.29 ± 0.44	2.13 ± 0.38	−0.16	−0.25 to −0.07	0.001
Environmental and interpersonal causes	2.06 ± 0.35	1.89 ± 0.36	−0.18	−0.26 to −0.09	< 0.001
Ward practices and systems	2.24 ± 0.31	2.08 ± 0.36	−0.16	−0.25 to −0.08	< 0.001
Management of aggression	1.89 ± 0.38	1.79 ± 0.32	−0.10	−0.18 to −0.02	0.010

Note: Lower mean scores indicate stronger agreement with statements about aggression and its management.

SD, standard deviation; CI, confidence interval.

**FIGURE 1:**
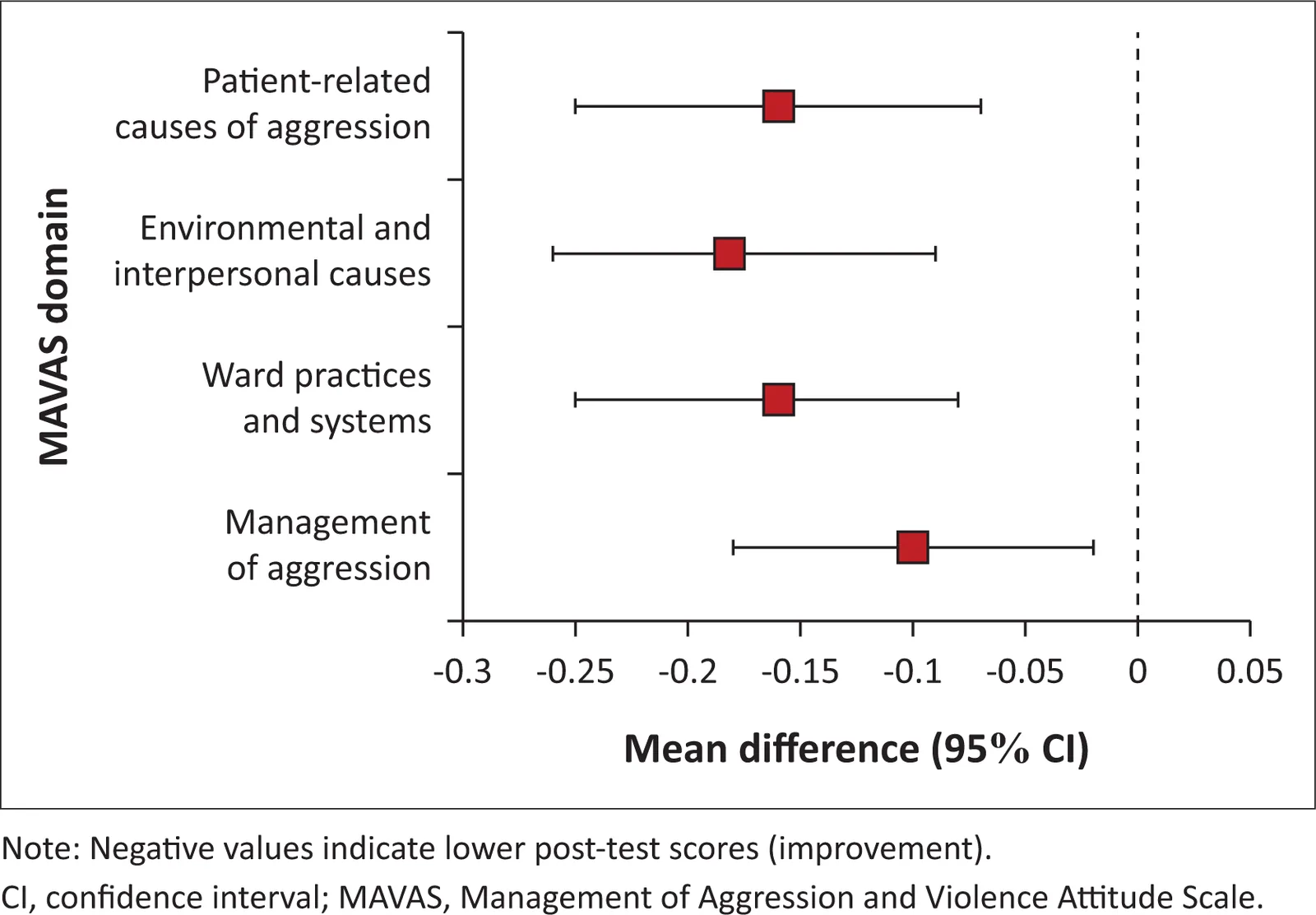
Ninety-five per cent mean differences in Management of Aggression and Violence Attitude Scale domain scores pre- and post-intervention.

Figure 1 presents the mean post–pre differences in MAVAS domain scores, with 95% CIs. All mean differences were negative, and none of the CIs crossed zero, showing consistent improvement across domains. The largest change occurred in environmental and interpersonal causes (−0.18; 95% CI: −0.26 to −0.09), followed by patient-related causes (−0.16; 95% CI: −0.25 to −0.07), ward practices and systems (−0.16; 95% CI: −0.25 to −0.08), and management of aggression (−0.10; 95% CI: −0.18 to −0.02). Because lower MAVAS scores represent stronger agreement with positive statements, these findings suggest improved attitudes towards the recognition and management of aggression post-intervention.

### Predictors of attitude change

#### Gender

When stratified by gender, no significant differences were observed in most domains. However, in the ward practices and systems domain, males demonstrated a larger reduction (−0.30 ± 0.34) than females (−0.05 ± 0.42; *p* = 0.003) (Table 3). This suggests that male participants showed greater change in recognising systemic factors related to aggression. Regression analysis identified female gender as a significant independent predictor of attitude improvement in the environmental and interpersonal causes of aggression domain (*B* = −0.259; *p* = 0.003; 95% CI: −0.427 to −0.090), indicating stronger post-intervention shifts in recognising situational and contextual contributors to aggression among female participants.

**TABLE 3:** Comparison of the change in Management of Aggression and Violence Attitude Scale domain scores by gender.

Domain	Female mean Δ (± SD)	Male mean Δ (± SD)	Mean difference	95% CI of the difference	*p*-value
Patient-related causes	−0.20 ± 0.43	−0.11 ± 0.46	−0.09	−0.28 to 0.10	0.332
Environmental and interpersonal	−0.19 ± 0.43	−0.16 ± 0.39	−0.03	−0.20 to 0.15	0.777
Ward practices and systems	−0.05 ± 0.42	−0.30 ± 0.34	0.25	0.08 to 0.41	**0.003**
Management of aggression	−0.14 ± 0.39	−0.06 ± 0.35	−0.08	−0.24 to 0.07	0.291

Note: Bold values indicate statistical significance at *p* < 0.05.

SD, standard deviation; CI, confidence interval.

* , Delta, change score calculated as post-intervention minus pre-intervention (negative values indicate stronger agreement after intervention).

#### Years of experience

Multiple linear regression analysis was conducted to identify independent predictors of changes in MAVAS domain scores while controlling for potential confounders. In this model, the unstandardised regression coefficient (*B*) represents the expected change in attitude score associated with each predictor variable, while the standardised coefficient (*β*) allows comparison of the relative strength of predictors. This analysis adds value by demonstrating which participant characteristics independently influenced attitude change beyond simple group comparisons.

Comparisons by years of experience revealed a significant difference in the patient-related causes domain (*p* = 0.030). Participants with more than 5 years of experience showed larger score reductions (−0.25 ± 0.38) than those with 1–5 years of experience (−0.05 ± 0.49), suggesting greater shifts in recognising patient-related contributors to aggression. Regression analysis confirmed that years of experience independently predicted changes in this domain (*B* = −0.227; *p* = 0.023; 95% CI: −0.422 to −0.032) (Table 4).

**TABLE 4:** Multiple linear regression predicting change in patient-related causes of aggression.

Predictor	*B*	SE	*β*	*t*-value	*p*-value	95% CI for *B*
Age (years)	0.003	0.006	0.06	0.53	0.599	−0.009 to 0.016
Gender (Female vs. Male)	0.132	0.094	0.15	1.40	0.165	−0.055 to 0.320
Ranking (Senior vs. Junior)	−0.076	0.14	−0.06	−0.54	0.589	−0.355 to 0.203
Years of working (> 5 vs. 1–5)	−0.227	0.098	−0.26	−2.32	**0.023**	−0.422 to −0.032

Note: Dependent variable: Change score (Post – Pre); negative values indicate stronger agreement post-intervention.

Significant values are shown in bold.

*B*, unstandardised regression coefficient; SE, standard error; *β*, standardised regression coefficient; *t*, test statistic; CI, confidence interval.

#### Ranking

No statistically significant differences were observed between junior and senior staff across any MAVAS domains (*p* > 0.05). Both groups showed comparable improvements in attitudes towards patient-related, environmental, and management aspects of aggression.

### A summary of key findings

Overall, the educational intervention significantly improved participants’ attitudes towards recognising and managing aggressive behaviour in hospital settings. Improvements were consistent across all MAVAS domains, with greater effects among female and more experienced staff. Age and professional ranking did not significantly influence outcomes. These results support the effectiveness of structured training programmes in enhancing staff attitudes towards de-escalation and aggression management.

## Discussion

Baseline MAVAS scores indicated that participants did not consistently endorse patient-centred and contextual explanations for aggression, suggesting scope for improvement in attitudes towards aggression management. While the scale does not provide cut-off values to define adequacy, these findings highlight the relevance of targeted training in this group.

Post-intervention scores demonstrated statistically significant shifts towards more favourable attitudes across all MAVAS domains. However, statistical significance alone does not indicate that acceptable or optimal attitudinal levels were achieved. These findings should therefore be interpreted as relative improvements rather than definitive attainment of best-practice standards.

Female participants exhibited greater changes in environmental and interpersonal causes of aggression, while staff with more than 5 years of experience demonstrated larger shifts in recognising patient-related causes. Age and professional rank did not significantly influence outcomes, indicating that the intervention benefited staff across hierarchical and demographic categories.

These findings are consistent with prior international and South African research demonstrating that de-escalation training positively influences staff attitudes towards aggression management, which, in other studies, has been associated with improved outcomes and reduced reliance on coercive measures, although such outcomes were not directly assessed in this study.^5,23,24,25,26,33,36^ Notably, the results indicate that non-clinical personnel, such as hospital security officers, can achieve outcomes comparable to those of clinical staff when provided with evidence-based training.^14,19,20^

The observed improvements across MAVAS attitude domains align with the biopsychosocial model, which conceptualises aggression as an interaction among behavioural, environmental, and systemic factors.^2,6,7^ Enhanced agreement with patient-related explanations suggests a shift towards empathy, contextual understanding, and non-punitive attitudes, consistent with Price and Baker^23^ and Richmond et al.^25^ The greater reduction in ward-practice-related scores among male participants may reflect their frequent deployment in high-intensity areas, such as psychiatric or casualty units, where aggression is closely linked to institutional stressors.^10,13,37^ Similarly, greater improvements among more experienced staff support prior research indicating that professional maturity enhances adaptability and reflective practice.^6,36^

These findings also reinforce NICE guidance, which recommends de-escalation as the first-line response to agitation in healthcare settings.^22^ The success of this intervention at CMH demonstrates that even within low-resource, high-risk environments, structured non-pharmacological strategies can improve staff attitudes towards the recognition and management of aggression.^19,26,33^

A key strength of this study is its ecological validity: the intervention was implemented in a real-world hospital setting frequently affected by patient aggression. The inclusion of nearly all available security personnel ensured representative data, and the use of the MAVAS enabled conceptual comparison with international studies that have employed the same validated instrument, although no direct statistical comparison with international datasets was undertaken in this study.^12^ In addition, the interactive, scenario-based format aligns with best practices in adult learning and aggression management education.^25,34,36^

Several limitations should be considered when interpreting these findings. Firstly, the single-group pre-test–post-test design without a control group limits causal inference, and improvements in attitudes cannot be attributed exclusively to the intervention. In addition, although MAVAS has been widely used internationally, the absence of direct comparative analyses limits quantitative cross-study comparisons. Secondly, attitudes were assessed using a self-report instrument, which may be subject to social desirability bias and does not directly measure behavioural change or clinical competence. Thirdly, although the MAVAS is a validated instrument, the isiXhosa version used in this study has not undergone formal psychometric validation. While forward translation and review by bilingual mental health professionals were undertaken to ensure conceptual equivalence, measurement error cannot be excluded. Fourthly, the short follow-up period precluded assessment of the durability of attitudinal changes over time. Finally, the relatively small number of senior staff may have limited the statistical power of subgroup analyses. Furthermore, a substantial proportion of eligible participants declined to participate (60 out of 170), which may introduce selection bias and limit the generalisability of the findings. Non-participation may have been influenced by operational demands, shift constraints, and varying levels of staff engagement. This has important implications for the implementation of similar training programmes, as high refusal rates could hinder scalability and effectiveness in routine practice. Future initiatives should consider strategies such as protected training time, leadership endorsement, and integration of training into routine staff development programmes to improve participation and sustainability. Future studies should incorporate controlled designs, validated translated instruments, and behavioural or observational outcome measures to strengthen evidence of effectiveness.

The results underscore the need to institutionalise structured de-escalation training as a core competency for hospital security personnel. Integrating such programmes into orientation and continuous professional development can reduce violent incidents, strengthen inter-professional collaboration, and enhance patient safety.^16,22,33,25^ Joint training for security and clinical staff is recommended, as interdisciplinary collaboration improves communication, aligns safety responses, and reduces role conflict.^15,37,38^ Furthermore, hospitals should implement refresher courses, monitoring systems, and mentorship initiatives to sustain skill retention and adherence to non-coercive practices.^17,24^

At a policy level, embedding de-escalation training within South Africa’s occupational and mental health safety frameworks would align national standards with global best practices, including the World Health Organization (WHO) World Report on Violence and Health and NICE guidelines.^5,22^ Addressing the shortage of trained personnel capable of safely managing aggression in psychiatric and emergency settings remains an urgent national priority.^14,16,37^ Future research should include controlled, longitudinal designs to evaluate sustained behavioural and cultural outcomes. In addition, qualitative studies could provide deeper insights into the lived experiences of security personnel and barriers to effective implementation.

## Conclusion

This study demonstrated that structured de-escalation training produced significant positive shifts in hospital security personnel’s attitudes towards the causes and management of aggression. While these changes reflect meaningful attitudinal improvement, further training and evaluation using behavioural or observational measures are required to determine whether such shifts translate into sustained practice change.
